# Differential hydroxylation efficiency of the two non-heme carotene hydroxylases: DcBCH1, rather than DcBCH2, plays a major role in carrot taproot

**DOI:** 10.1093/hr/uhac193

**Published:** 2022-08-30

**Authors:** Tong Li, Jie-Xia Liu, Yuan-Jie Deng, Ao-Qi Duan, Hui Liu, Fei-Yun Zhuang, Ai-Sheng Xiong

**Affiliations:** State Key Laboratory of Crop Genetics and Germplasm Enhancement, Ministry of Agriculture and Rural Affairs Key Laboratory of Biology and Germplasm Enhancement of Horticultural Crops in East China, College of Horticulture, Nanjing Agricultural University, 1 Weigang, Nanjing, 210095, China; State Key Laboratory of Crop Genetics and Germplasm Enhancement, Ministry of Agriculture and Rural Affairs Key Laboratory of Biology and Germplasm Enhancement of Horticultural Crops in East China, College of Horticulture, Nanjing Agricultural University, 1 Weigang, Nanjing, 210095, China; State Key Laboratory of Crop Genetics and Germplasm Enhancement, Ministry of Agriculture and Rural Affairs Key Laboratory of Biology and Germplasm Enhancement of Horticultural Crops in East China, College of Horticulture, Nanjing Agricultural University, 1 Weigang, Nanjing, 210095, China; State Key Laboratory of Crop Genetics and Germplasm Enhancement, Ministry of Agriculture and Rural Affairs Key Laboratory of Biology and Germplasm Enhancement of Horticultural Crops in East China, College of Horticulture, Nanjing Agricultural University, 1 Weigang, Nanjing, 210095, China; State Key Laboratory of Crop Genetics and Germplasm Enhancement, Ministry of Agriculture and Rural Affairs Key Laboratory of Biology and Germplasm Enhancement of Horticultural Crops in East China, College of Horticulture, Nanjing Agricultural University, 1 Weigang, Nanjing, 210095, China; Key Laboratory of Horticultural Crop Biology and Germplasm Innovation, Ministry of Agriculture; Institute of Vegetables and Flowers, Chinese Academy of Agricultural Science, Beijing 100081, China; State Key Laboratory of Crop Genetics and Germplasm Enhancement, Ministry of Agriculture and Rural Affairs Key Laboratory of Biology and Germplasm Enhancement of Horticultural Crops in East China, College of Horticulture, Nanjing Agricultural University, 1 Weigang, Nanjing, 210095, China

## Abstract

Carotene hydroxylase plays an important role in catalyzing the hydroxylation of carotene to xanthopylls, including two types: non-heme carotene hydroxylase (BCH type) and heme-containing cytochrome P450 hydroxylase (P450 type). Two BCH-encoding genes were annotated in the carrot genome. However, the role of BCHs and whether there are functional interactions between the duplicated BCHs in carrot remains unclear. In this study, two BCH encoding genes, *DcBCH1* and *DcBCH2*, were cloned from carrot. The relative expression level of *DcBCH1* was much higher than that of *DcBCH2* in carrot taproots with different carotene accumulation levels. Overexpression of *DcBCH1* in ‘KRD’ (high carotene accumulated) carrot changed the taproot color from orange to yellow, accompanied by substantial reductions in α-carotene and β-carotene. There was no obvious change in taproot color between transgenic ‘KRD’ carrot overexpressing *DcBCH2* and control carrot. Simultaneously, the content of α-carotene in the taproot of *DcBCH2-*overexpressing carrot decreased, but the content of β-carotene did not change significantly in comparison with control carrot. Using the CRISPR/Cas9 system to knock out *DcBCH1* in ‘KRD’ carrot lightened the taproot color from orange to pink-orange; the content of α-carotene in the taproot increased slightly, while the β-carotene content was still significantly decreased, compared with control carrot. In *DcBCH1*-knockout carrot, the transcript level of *DcBCH2* was significantly increased. These results indicated that in carrot taproot, DcBCH1 played the main function of BCH enzyme, which could hydroxylate α-carotene and β-carotene; DcBCH1 and DcBCH2 had functional redundancy, and these two DcBCHs could partially compensate for each other.

## Introduction

Carotenoids are lipid-soluble terpenoids composed of an isoprene skeleton, which exhibit bright colors including red, orange or yellow under visible light, due to the existence of multiple conjugated double bonds in their molecular structure [1–3]. As one of the important coloring substances, carotenoids endow plants’ different organs such as flowers, fruits, seeds, roots, etc. with rich colors, and also play important roles in the process of plants resisting adversity [3–6]. In addition, carotenoids and their derivatives play a vital role in maintaining human health and delaying body aging [7, 8]. The most widely distributed carotenoids mainly include the hydrocarbon carotenes, such as lycopene, β-carotene, and α-carotene, and the xanthophylls, such as lutein, zeaxanthin, violaxanthin, neoxanthin, and capsanthin. In plants, the biosynthetic pathway of carotenoids has been extensively studied and well-characterized, and most of the enzymes and genes have been identified [3]. Carotene hydroxylases are key enzymes in the production of xanthophylls, which catalyze the hydroxylation of the β- and ε-ring at the 3,3′ positions of carotene (α-carotene and β-carotene) to produce lutein (α-carotene-derived xanthophyll)and zeaxanthin (β-carotene-derived xanthophyll); the latter can be further modified to produce other xanthophylls. There are two different types of carotene hydroxylases in plants: the non-heme di-iron oxygenase (BCH, also known as BHY, HYD, or HYb) and heme-containing cytochromes P450 oxygenase (CYP97A, CYP97B, and CYP97C) [9–12].

BCH mainly hydroxylates the β-ring of carotene and is widely found in organisms such as plants, bacteria, and cyanobacteria [13–15]. Phylogenetic analysis revealed that duplication events of the *BCH* genes occurred in higher plants after monocotyledonous and dicotyledonous splits [16]. Some studies have shown that the BCH encoded by the duplicated genes have the same function, mainly act on β-carotene, and also have a hydroxylation effect on α-carotene containing a β-ring [17, 18]. In the pulp of transgenic orange (*Citrus sinensis*) that silenced the expression of the β-carotene hydroxylation gene (*Csβ-CHX*), the β-carotene content was increased 36-fold compared to control plant [19]. In wheat (*Triticum aestivum*), specifically blocking the expression of the endogenous *BCH* in endosperm resulted in a 10.5-fold increase in endosperm β-carotene levels [20]. In *Arabidopsis thaliana*, the proteins (β-hydroxylase 1 and β-hydroxylase 2) encoded by *β-OHase 1* and *β-OHase 2* can effectively catalyze the hydroxylation of the C3 position of carotene containing β-ring and had only weak activity on carotene containing ε-ring *in vitro*, while there were differences in the functional strength of the two proteins *in vivo* [14, 21]. Studies have also found that the functions of BCH encoded by duplicated genes in some species are not completely consistent. For example, BCH1 and BCH2 of maize (*Zea mays*) showed differences in enzymatic function *in vitro* [22]. In addition, *BCH* genes in some higher plants exhibited gene-specific expression patterns and contributed to different carotenoid levels in a tissue-specific manner [23]. In tomato (*Solanum lycopersicum*), the *bch2* (*CrtR-b2*) mutant produced a colorless phenotype in petals, but had no effect on lutein biosynthesis in leaves [17]. In pepper (*Capsicum annuum*), mutations in the gene encoding β-carotene hydroxylase (CHY2) led to the accumulation of β-carotene in pepper fruit, which changed the pepper fruit color from red to orange [24].

Carrot (*Daucus carota*) is one of the top ten vegetable crops in the world; its taproot color is closely related to the composition and accumulation of carotenoids [25–27]. Orange carrot taproot accumulates large amounts of α-carotene and β-carotene [27]. There were two BCH encoding genes annotated in the carrot genome database [28]. The role of these two BCHs and what is the relationship between them, such as functional complementarity or redundancy, in carrot remains unclear. Here, we isolated the two BCH encoding genes, *DcBCH1* and *DcBCH2*, from carrot. The relative expression level of *DcBCH1* was much higher than *DcBCH2* in the taproot of two carrot cultivars with different carotene (α-carotene and β-carotene) accumulation. We overexpressed *DcBCH1* and *DcBCH2* in ‘KRD’ (high carotene accumulation) and investigated carotene content in transgenic carrot taproot, and further knocked out *DcBCH1* in ‘KRD’ to evaluate the hydroxylation functions of carotene by these two BCH enzymes (DcBCH1 and DcBCH2) and its relationship.

## Results

### Isolation and analysis of *DcBCH1* and *DcBCH2*

**Figure 1:**
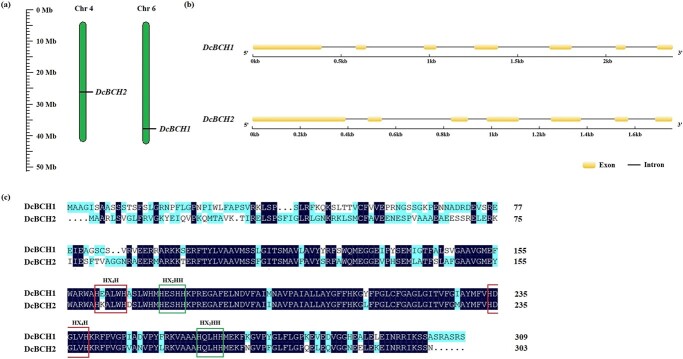
Bioinformatics analysis of *DcBCH1* and *DcBCH2*. (**a**) Chromosomal location of *DcBCH1* and *DcBCH2*. (**b**) The gene structure of *DcBCH1* and *DcBCH2*. (**c**) Alignment of DcBCH1 and DcBCH2 amino acid sequences from ‘KRD’: the red box indicates the ‘HX_4_H’ domain; the blue box indicates the ‘HX_2_HH’ domain.

Based on the carrot genome database, a total of two BCH genes (*DcBCH1* and *DcBCH2*) were annotated. *DcBCH1* and *DcBCH2* were located on chromosomes 6 and 4 in carrot, respectively, with the same structure both containing seven exons (Fig. 1a and b). The full-length open reading frames (ORFs) of *DcBCH1* and *DcBCH2* isolated from ‘KRD’ were 930 bp and 912 bp, encoding 309 and 303 amino acids, respectively (Figs S1 and 2, see online supplementary material). *DcBCH1* and *DcBCH2* from ‘KRD’ and the genome database differ by 7 and 19 sites different at the nucleotide level, two and seven sites different at the amino acid level, respectively. In addition, the predicted molecular weights and isoelectric points of DcBCH1 and DcBCH2 proteins from ‘KRD’ were 34.2 kDa and 33.89 kDa, 9.04 and 8.66, respectively.

**Figure 2:**
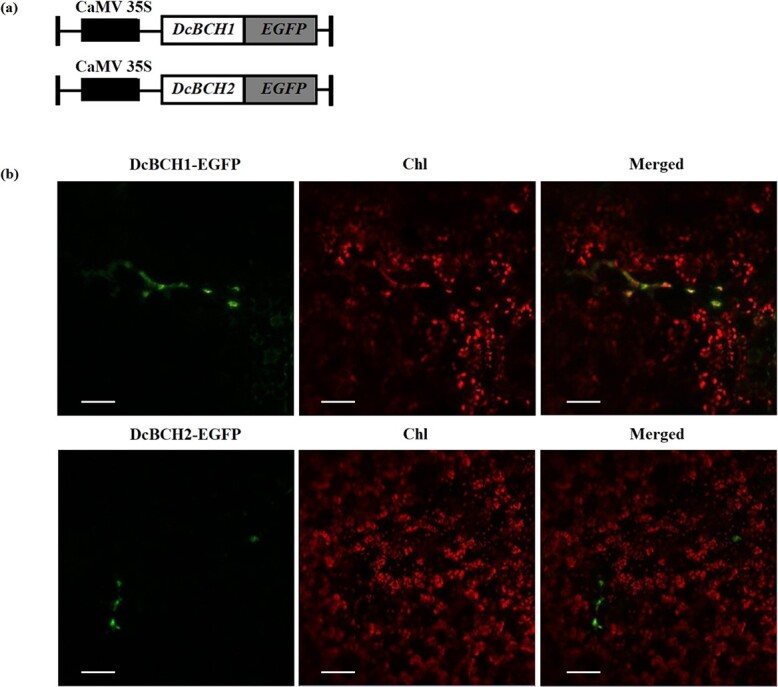
Subcellular localization of DcBCH1 and DcBCH2. (**a**) Diagram of the DcBCH1-EGFP and DcBCH2-EGFP construct; (**b**) Subcellular localization of DcBCH1 and DcBCH2 in tobacco mesophyll cells. Chl: chloroplast. Scale bar = 50 μm.

The similarity between DcBCH1 and DcBCH2 protein sequences was 65.61% (Fig. 1c). Further sequence multiple alignment of DcBCH1 and DcBCH2 with BCHs from other plant species found that DcBCH1 and DcBCH2 contained four conserved histidine domains: ‘HX_4_H’ (HE/KALWH), ‘HX_2_HH’ (HESHH), ‘HX_4_H’ (HDGLVH), and ‘HX_2_HH’ (HQLHH) to ensure the catalytic activity of carotene hydroxylase (Fig. 1c; Fig. S3, see online supplementary material). Evolutionary analysis results showed that DcBCH1 and DcBCH2 were most closely related to BCH1 from *Apium graveolens* (AgBCH1) (Fig. S4, see online supplementary material).

### Subcellular localization of DcBCH1 and DcBCH2

To analyse the subcellular localization of DcBCH1 and DcBCH2, the recombinant vectors *DcBCH1-EGFP* and *DcBCH2-EGFP* were introduced individually into tobacco mesophyll cells. The results showed that the green fluorescence signals of DcBCH1-EGFP and DcBCH2-EGFP were distributed in the chloroplast and overlapped with the red fluorescence signal of the chloroplast, indicating that both DcBCH1 and DcBCH2 were localized in the chloroplast (Fig. 2).

### Expression analysis of *DcBCH1* and *DcBCH2* in carrot taproot

**Figure 3:**
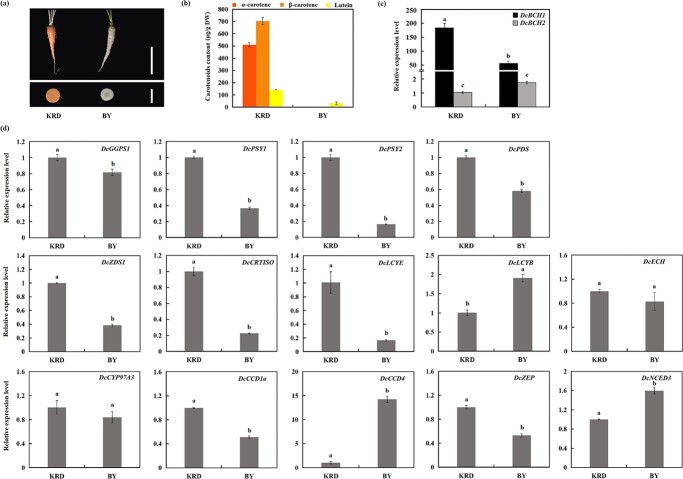
The relative expression levels of carotenoid metabolism related genes in the taproot of ‘KRD’ and ‘BY’. (**a**–**b**) The phenotypes (**a**) and carotenoids (α-carotene, β-carotene and lutein) (**b**) content of ‘KRD’ and ‘BY’ taproot, long scale = 5 cm, short scale = 1.3 cm; (**c**) The relative expression levels of *DcBCH1* and *DcBCH2* in ‘KRD’ and ‘BY’ taproot were calculated using the expression level of *DcBCH2* in ‘KRD’ taproot as the standard (set the value as 1), the letters above the bars indicate significant differences between the gene expression level (*P* < 0.05, according to Tukey’s multiple range test); (**d**) The relative expression levels of these carotenoid metabolism related genes in ‘KRD’ and ‘BY’ taproots were calculated using each gene expression level in ‘KRD’ taproot as the standard (set the value as 1), the letters above the bars indicate significant differences at *P* < 0.05 level, according to Student’s *t* test. Bars represent mean standard deviation (SD).

The contents of α-carotene, β-carotene and lutein in the taproot of two carrot cultivars with different taproot colors were determined. As shown in Fig. 3a and b, a large amount of α-carotene and β-carotene accumulated in the taproot of the orange carrot (‘KRD’), while the taproot of ‘BY’ was pale yellow and nearly white, only a small amount of lutein was detected.

RT-qPCR analysis showed that the expression level of *DcBCH1* in the taproot of ‘KRD’ and ‘BY’ was significantly higher than that of *DcBCH2*. In addition, the expression level of *DcBCH1* in ‘KRD’ was significantly higher than that in ‘BY’; *DcBCH2* expression level was not significantly different between ‘KRD’ and ‘BY’ (Fig. 3c). Among other 14 carotenoid metabolism-related genes, *DcGGPS1*, *DcPSY1*, *DcPSY2*, *DcPDS*, *DcZDS1*, *DcCRTISO*, *DcLCYE*, *DcZEP*, and *DcCCD1a* showed a lower transcript level in ‘BY’ than that in ‘KRD’; while the transcript level of *DcLCYB*, *DcCCD4*, and *DcNCED3* in ‘BY’ was higher than that in ‘KRD’ (Fig. 3d).

### Generation of transgenic carrot plants overexpressing *DcBCH1* and *DcBCH2*

**Figure 4:**
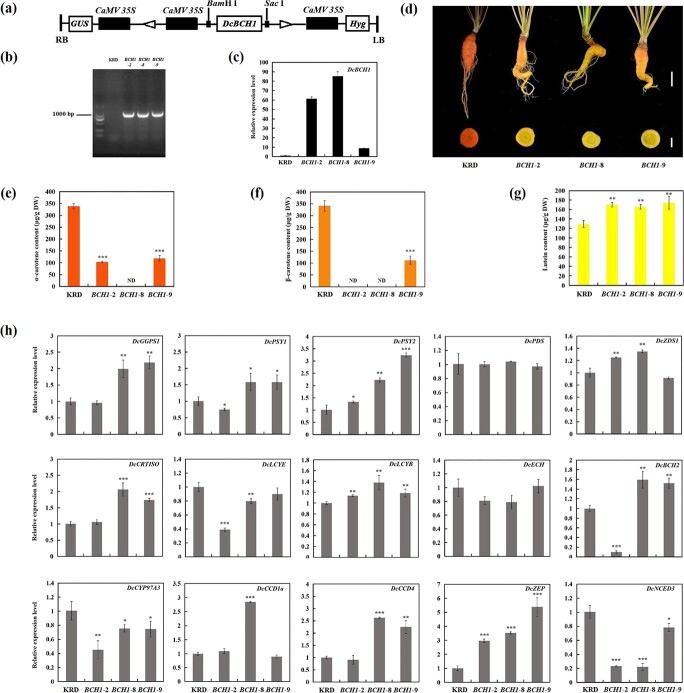
Effects of overexpression of *DcBCH1* on carotenoid accumulation in carrot taproot. (**a**) Diagram of the *DcBCH1*-overexpressing construct. (**b**) PCR amplification of *DcBCH1* from control carrot (KRD) and *DcBCH1*-overexpressing transgenic carrot (*BCH1*–2, *BCH1*–8, and *BCH1*–9) cDNA. (**c**) Relative expression level of *DcBCH1* in three *DcBCH1*-overexpressing transgenic carrot lines (*BCH1*–2, *BCH1*–8 and *BCH1*–9). (**d**) Taproot phenotype of control carrot (KRD) and *DcBCH1*-overexpressing transgenic carrot, long scale bar = 2 cm, short scale bar = 1 cm. (**e**–**g**) α-carotene (**e**), β-carotene (**f**), and lutein (**g**) content in taproot of control carrot (KRD) and *DcBCH1*-overexpressing transgenic carrot, ND represents not detected. (h) Relative expression levels of carotenoid metabolism related genes in the taproot of control carrot (KRD) and *DcBCH1*-overexpressing transgenic carrot. Bars represent mean standard deviation (SD). The statistical significance of the measurements between transgenic carrot (*BCH1*–2, *BCH1*–8, and *BCH1*–9) and the control carrot (KRD) was determined using Student’s *t* test (^*^P < 0.05; ^**^P < 0.01; ^***^P < 0.001).

**Figure 5:**
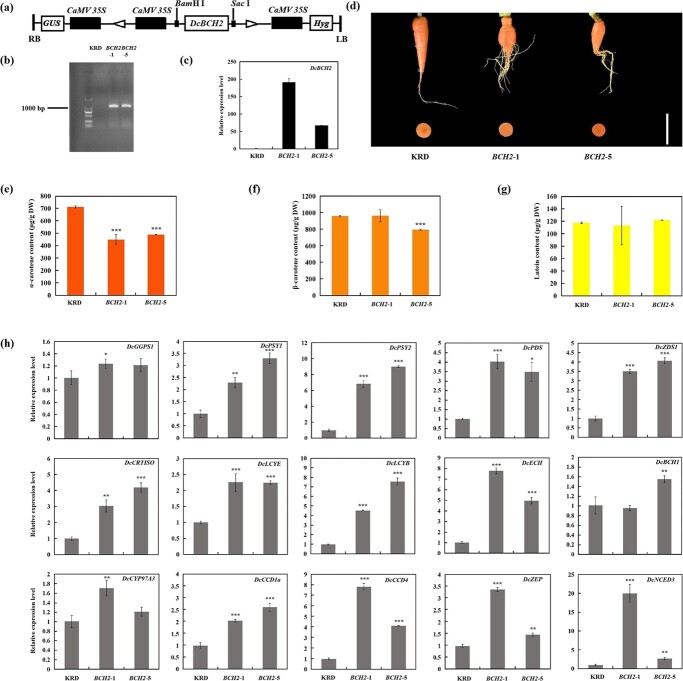
Effects of overexpression of *DcBCH2* on carotenoid accumulation in carrot taproot. (**a**) Diagram of the *DcBCH2*-overexpressing construct. (**b**) PCR amplification of *DcBCH2* from control carrot (KRD) and *DcBCH2*-overexpressing transgenic carrot (*BCH2*–1 and *BCH2*–5) cDNA. (**c**) Relative expression level of *DcBCH2* in two *DcBCH2*-overexpressing transgenic carrot lines (*BCH2*–1 and *BCH2*–5). (**d**) Taproot phenotype of control carrot (KRD) and *DcBCH2*-overexpressing transgenic carrot, scale bar = 5 cm. (**e**-**g**) α-carotene (**e**), β-carotene (**f**), and lutein (**g**) content in taproot of control carrot (KRD) and *DcBCH2*-overexpressing transgenic carrot. (**h**) Relative expression levels of carotenoid metabolism related genes in the taproot of control carrot (KRD) and *DcBCH2-*overexpressing transgenic carrot. Bars represent mean standard deviation (SD). The statistical significance of the measurements between transgenic carrot (*BCH2*–1 and *BCH2*–5) and the control carrot (KRD) was determined using Student’s *t* test (^*^P < 0.05; ^**^P < 0.01; ^***^P < 0.001).

Transgenic carrots overexpressing *DcBCH1* and *DcBCH2* were generated to investigate the effect of up-regulation of *DcBCH1* and *DcBCH2* expression on the accumulation of carotenoids in carrot taproot, and differences in function between DcBCH1 and DcBCH2. After verification by PCR amplification (Figs 4b and 5b), transgenic carrot lines overexpressing *DcBCH1* (*BCH1*–2, *BCH1*–8, and *BCH1*–9) and *DcBCH2* (*BCH2*–1 and *BCH2*–5) were selected to further analysis. Transcript level assessment determined by RT-qPCR revealed that the expression level of *DcBCH1* was the highest in the *BCH1*–8; and the *BCH2*–5 had the highest transcript level of *DcBCH2* (Figs 4c and 5c).

### Effects of *DcBCH1* overexpression on carotenoids content and expression profiles of metabolism-related genes in carrot taproot

The taproot phenotype of three *DcBCH1*-overexpressing transgenic carrot lines (*BCH1*–2, *BCH1*–8, and *BCH1*–9) and control carrot (KRD) were observed. As shown in Fig. 4d, there was a clear difference in the taproot color between the transgenic and control carrots, the taproot of transgenic carrot hosting *DcBCH1* gene was yellow, while the control carrot was orange.

The accumulation of carotenoids in the taproot of the above carrot materials was further determined. Compared with control carrot (KRD), the content of α-carotene in transgenic carrot taproot was reduced by 65%–70% (*BCH1*–9 and *BCH1*–2), and the accumulation of α-carotene was not detected in *BCH1*–8 (Fig. 4e). In the taproot of *BCH1*–2 and *BCH1*–8, the accumulation of β-carotene was not detected, and the β-carotene content in *BCH1*–9 was significantly lower than that in control carrot (Fig. 4f). In addition, the lutein content in transgenic carrot taproot was slightly higher than that in the control carrot (Fig. 4g).

To further analyse the changes in carotenoid accumulation, the relative expression levels of carotenoid metabolism-related genes in the taproot of control carrot (KRD) and *DcBCH1*-overexpressing transgenic carrot were measured. As shown in Fig. 4h, in transgenic carrot (*BCH1*–8 and *BCH1*–9), the relative expression levels of *DcGGPS1*, *DcPSY1*, *DcCRTISO*, and *DcCCD4* were increased to different degrees compared with control carrot. The expression levels of *DcPDS* and *DcECH* were similar between control carrot and transgenic carrot. Compared with the control carrot, the relative expression levels of *DcPSY2*, *DcLCYB*, and *DcZEP* were increased in the three transgenic carrot lines (*BCH1*–2, *BCH1*–8, and *BCH1*–9), and the expression level of *DcZEP* in three transgenic carrot lines was 3 ~ 5.4 times higher than that in the control carrot. On the contrary, the expression levels of *DcLCYE*, *DcCYP97A3*, and *DcNCED3* in three transgenic carrot lines were reduced in comparison with control carrot. In addition, in *BCH1*–8 and *BCH1*–9, the expression level of *DcBCH2* was about 1.6 times higher than that in control carrot; while the expression level of *DcBCH2* in *BCH1*–2 decreased by 87%, compared with control carrot.

### Effects of *DcBCH2* overexpression on carotenoids content and expression profiles of metabolism-related genes in carrot taproot

There was no obvious difference in taproot color between *DcBCH2-*overexpressing transgenic carrot (*BCH2*–1 and *BCH2*–5) and control carrot (KRD) (Fig. 5d). The content of α-carotene in taproot of the transgenic carrot was significantly lower than that of control carrot, decreasing by 37% (*BCH2*–1) and 31% (*BCH2*–5), respectively (Fig. 5e). Compared with the control carrot, the content of β-carotene in *BCH2*–5 was reduced by 17%, while the content of β-carotene in *BCH2*–1 was not significantly changed (Fig. 5f). In addition, lutein content in taproot between transgenic and control carrot was no significant difference (Fig. 5g).

Further analysis of the relative expression levels of carotenoid metabolism-related genes in the taproot of transgenic and control carrot (KRD). As shown in Fig. 5h, among the 15 genes detected (*DcGGPS1*, *DcPSY1*, *DcPSY2*, *DcPDS*, *DcZDS1*, *DcCRTISO*, *DcLCYE*, *DcLCYB*, *DcECH*, *DcBCH1*, *DcCYP97A3*, *DcCCD1a*, *DcCCD4*, *DcZEP*, and *DcNCED3*), except for *DcBCH1* and *DcGGPS1*, the other 13 genes expression levels in transgenic carrot taproot were significantly higher than those in control carrot. The relative expression levels of *DcLCYB* and *DcECH* in *BCH2*–1 and *BCH2*–5 were 4.6- and 7.6-fold, 7.8- and 5-fold higher than those in control carrot, respectively. Compared with the control carrot, the expression level of *DcCYP97A3* was increased by 1.7-fold in *BCH2*–1, while the expression in *BCH2*–5 was not significantly different from that in control carrot. There was no significant difference in the relative expression level of *DcBCH1* between *BCH2*–1 and control carrot, while in *BCH2*–5, the expression level of *DcBCH1* was 1.6-fold higher than that in control carrot.

### Generation of *DcBCH1* knockout mutant carrot by CRISPR/Cas9

**Figure 6:**
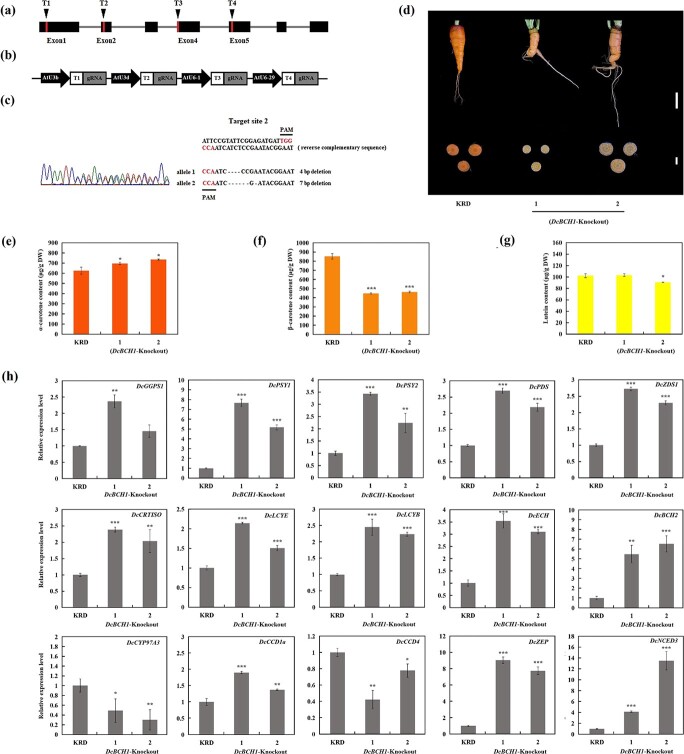
Effects of *DcBCH1* knockout on carotenoid accumulation in carrot taproot. (**a**) The positions of the four target sites on the *DcBCH1* gene structure. (**b**) Structure diagram of the assembly of four sgRNA expression cassettes. (**c**) Sequence peak map of successful *DcBCH1* editing sites in *DcBCH1*-Knockout carrot. (**d**) Taproot phenotype of control carrot (KRD) and *DcBCH1*-Knockout carrot, long scale bar = 2 cm, short scale bar = 1 cm. (**e**-**g**) α-carotene (**e**), β-carotene (**f**) and lutein (**g**) content in taproot of control carrot (KRD) and *DcBCH1*-Knockout mutant carrot. (**h**) Relative expression levels of carotenoid metabolism related genes in the taproot of control carrot (KRD) and *DcBCH1*-Knockout mutant carrot. Bars represent mean standard deviation (SD). The statistical significance of the measurements between *DcBCH1*-Knockout carrot and the control carrot (KRD) was determined using Student’s *t* test (^*^P < 0.05; ^**^P < 0.01; ^***^P < 0.001).

It was observed that overexpression of *DcBCH1* significantly reduced carotene accumulation in transgenic carrot taproot and changed the color of taproot. Therefore, we further utilized the CRISPR/Cas9-mediated genome editing system to knock out *DcBCH1* in ‘KRD’. Based on the sequence of *DcBCH1*, four target sites (T1, T2, T3, and T4) located on the first, second, fourth, and fifth exons were selected (Fig. 6a). The four target site sgRNAs driven by the promoters AtU3b, AtU3d, AtU6–1, and AtU6–29, respectively, were assembled and inserted into the *pYLCRISPR/Cas9Pubi-H* vector to obtain a knockout expression vector (Fig. 6b). Genetic transformation of carrot was carried out using *Agrobacterium*-mediated method. The fragments containing the target site were amplified from the obtained resistant plant gDNA by PCR using specific primers, and then sequenced directly to detect the mutation of the target site. The direct sequencing results showed that the target site 2 (targeted by AtU3d-driven sgRNA) was mutated in the obtained transgenic resistant plant, and there were no changes in the other three target sites (target sites 1, 3, and 4). Further analysis showed that biallelic mutation occurred in target site 2 (Fig. 6c).

### Effects of *DcBCH1* knockout on carotenoids content and expression profiles of metabolism-related genes in carrot taproot

The taproot phenotypes of two plants of *DcBCH1* mutant carrot (*DcBCH1*-Knockout-1 and *DcBCH1*-Knockout-2) were observed. As shown in Fig. 6d, the taproot color of *DcBCH1*-Knockout carrot became lighter, showing a pink-orange phenotype. Carotenoids content determination results showed that the content of α-carotene in *DcBCH1*-Knockout carrot taproot was slightly higher than that of control carrot (Fig. 6e). In *DcBCH1*-Knockout carrot taproot, the content of β-carotene was significantly reduced, which was about 45% lower than that of control carrot (Fig. 6f). In addition, the lutein content of *DcBCH1*-Knockout and control carrot was not significantly different or slightly lower than that of control carrot (Fig. 6g).

The expression level of *DcBCH2* in *DcBCH1*-Knockout carrot was 5.5-fold (*DcBCH1*-Knockout-1) and 6.6-fold (*DcBCH1*-Knockout-2) than that in control carrot (KRD), respectively. Compared with the control carrot, the expression levels of *DcPSY1*, *DcPSY2*, *DcPDS*, *DcZDS1*, *DcCRTISO*, *DcLCYE*, *DcLCYB*, *DcECH*, *DcZEP*, and *DcCCD1a* in *DcBCH1*-Knockout carrot were all up-regulated to varying degrees. *DcNCED3* transcript level in *DcBCH1*-Knockout carrot was 4 ~ 13.5-fold higher than that in control carrot. In addition, the expression of *DcCYP97A3* in *DcBCH1*-Knockout carrot was reduced by 51%–70% in comparison with the control carrot (Fig. 6h).

## Discussion

Most of higher plants contain two or more BCH (BHY) with high sequence similarity [17, 29]. In *A. thaliana*, two BCH encoding genes, *β-OHase 1* and *β-OHase 2*, were identified with 70% sequence similarity, and located on chromosomes 4 and 5, respectively [21]. In the maize genome, two functional *BCH* genes, *Zmbch1* and *Zmbch2* (located on chromosomes 2 and 10, respectively, with protein similarity of 76.6%), two pseudogenes of *BCH*, and more than two *BCH* homologous genes with unknown functions were annotated [22, 30]. Carrot, a biennial herb of the genus *Daucus* in the Apiaceae family, is an important root vegetable crop [31]. With the development of high-throughput sequencing technology, more and more genome sequences of some important plants belonging to the Apiaceae family have been completed [28, 32–34]. A total of two BCH encoding genes were annotated in the carrot genome, located on chromosomes 4 and 6, respectively [28]. We cloned these two BCH encoding genes from carrot (‘KRD’) and named them as *DcBCH1* and *DcBCH2*. The protein sequence similarity between DcBCH1 and DcBCH2 was 65.61%. Studies have confirmed that BCH proteins from different species contained the characteristic motifs, four conserved histidine domains (‘HX_4_H’, ‘HX_2_HH’, ‘HX_4_H’, and ‘HX_2_HH’) to ensure the normal activity of BCH [35, 36]. Sequence alignment results showed that both DcBCH1 and DcBCH2 contained four complete conserved histidine domains in the amino acid sequence.

In plants, the presence of isozymes often reflects the need for the same catalytic action in different subcellular compartments [37]. Plastids are the main site for carotenoid biosynthesis and storage in plants. Subcellular localization analysis confirmed that BCH from other species was localized in plastid (chloroplast) [38]. Our study showed that DcBCH1 and DcBCH2 were also located in the plastid (chloroplast). These results indicated that DcBCH1 and DcBCH2 had normal hydroxylase activity and played catalytic roles in the same subcellular location. We further detected the expression of *DcBCH1* and *DcBCH2* to preliminarily analyse whether there was a functional difference between DcBCH1 and DcBCH2. This is similar to previous research that the orange carrot taproots mainly accumulated a lot of α-carotene and β-carotene, and the nearly white carrot only accumulated little lutein or almost no carotenoids [25, 26, 39]. Gene expression is affected by many factors, such as materials, planting management, growth and development, environmental factors, etc. [18, 38, 40]. RT-qPCR analysis showed that many carotenoid metabolism-related genes expression levels in the orange carrot taproot were higher than those in the nearly white carrot taproot; both *DcBCH1* and *DcBCH2* were expressed in the orange and nearly white carrot taproots, and the relative expression level of *DcBCH1* was much higher than that of *DcBCH2*. These results indicated that DcBCH1 may play a major role in carrot taproot.

Overexpression of *DcBCH1* in carrot with high carotene (α-carotene and β-carotene) accumulation changed the color of the transgenic carrot taproot from orange (the phenotype of the control carrot) to yellow. Corresponding to the phenotypic results, the contents of α-carotene and β-carotene in transgenic carrot taproot were greatly reduced compared with those of the control carrot, especially the content of β-carotene. In transgenic tomato fruits hosting kiwifruit *AcBCH1*, the β-carotene content significantly decreased; in *AcBCH1* overexpressing transgenic kiwifruit leaves, the β-carotene content decreased and zeaxanthin content increased [41]. Overexpression of *chyB* in *A. thaliana* increased the contents of total carotenoids and xanthophyll such as zeaxanthin and violaxanthin, and decreased β-carotene content [42]. In *DcBCH1*-overexpressing transgenic carrot taproot, the lutein (α-carotene-derived xanthophyll) content was increased. These findings implied that the β-carotene-derived xanthophylls content may also be increased, and the decrease of α-carotene and β-carotene content was mainly due to the production of DcBCH1 hydroxylation products (α- and β-carotene-derived xanthophylls). There was no significant difference in taproot color between *DcBCH2-*overexpressing transgenic and control carrot. In two *DcBCH2*-overexpressing transgenic carrot lines taproot, the α-carotene content was significantly lower than that of control carrot; the content of β-carotene in *BCH2*–1 taproot did not change significantly, while in *BCH2*–5 taproot was decreased, compared to control carrot. In *A. thaliana*, *β-OHase 1* and *β-OHase 2* were expressed in all experiment tissues, and the *β-OHase 1* transcript level was 10–50 times higher than that of *β-OHase 2*. Both β-hydroxylase 1 (β-OHase 1) and β-hydroxylase 2 (β-OHase 2) were confirmed to effectively hydroxylate the β-ring, and had poor hydroxylation ability to substrates containing the ε-ring *in vitro* [21]. MpBHY can hydroxylate the β-ring of β-carotene and α-carotene, and promote the biosynthesis of zeaxanthin and lutein [43]. In *Escherichia coli* BL21 (DE3), CitHYb from sweet orange could catalyze the hydroxylation of the β-ring of β-carotene and α-carotene [18]. These results indicated that both DcBCH1 and DcBCH2 have the activity of hydroxylating β-ring *in vivo*.

During maize endosperm development, the expression profiles of *Zmbch1* and *Zmbch2* were consistent, but in *in vitro* enzyme activity experiments, ZmBCH1 could hydroxylate β-carotene into β-cryptoxanthin and zeaxanthin, while ZmBCH2 could only hydroxylate β-carotene to β-cryptoxanthin alone, and the overall activity of ZmBCH2 was lower than that of ZmBCH1 [22]. In three *DcBCH1-*overexpressing transgenic carrot lines, the *DcBCH2* transcript level in *BCH1*–2 was significantly lower, while in the other two transgenic lines (*BCH1*–8 and *BCH1*–9) was significantly higher in comparison with the control carrot. The expression level of *DcBCH2* in *BCH1*–8 (with the highest *DcBCH1* transcript level) was the highest. Correspondingly, only lutein accumulation was detected in *BCH1*–8 taproot, while α-carotene and β-carotene were not detected. In *BCH1*–9 (with the lowest *DcBCH1* transcript level), the *DcBCH2* expression level was slightly lower than *BCH1*–8; the accumulation of α-carotene and β-carotene was still detected in its taproot. In *BCH1*–2 (the *DcBCH1* transcript level was slightly lower than *BCH1*–8) taproot, the *DcBCH2* expression level was only 13% of control carrot, the accumulation of β-carotene was not detected; while the content of α-carotene was slightly lower than *BCH1*–9. Lycopene ε-cyclase (LCYE) and lycopene β-cyclase (LCYB) work together on all-*trans* lycopene to produce α-carotene and β-carotene, respectively. Silencing the expression of *LCYE* in sweet potato (*Ipomoea batatas*) by RNAi reduced the content of α-carotene and increased the content of β-carotene in storage roots [6]. The β-carotene content in the mature fruits of transgenic tomato hosting the tobacco *LCYB* was increased by five times in comparison with control plants, and exhibited an orange pigmentation phenotype [44]. Compared with control carrot, the expression level of *DcLCYB* was increased, while the expression level of *DcLCYE* was decreased to varying degrees, in the taproot of *DcBCH1*-overexpressing transgenic carrot, suggesting that overexpression of *DcBCH1* partially biased the carotenoid flux towards the β-carotene biosynthesis branch. In the taproot of *DcBCH2-*overexpressing transgenic carrot, the transcription levels of *DcLCYE* and *DcLCYB* were higher than those in control carrot. Among them, the transcription level of *DcLCYB* was higher in *BCH2*–5, which had lower *DcBCH2* transcription level and higher *DcBCH1* transcription level. Based on the above results, we speculated that when DcBCH1 and DcBCH2 coexisted, DcBCH1 played a role in hydroxylation of both α-carotene and β-carotene, and was more inclined to the hydroxylation of β-carotene, while DcBCH2 may play a complementary role to the function of DcBCH1, possibly more inclined to the hydroxylation of α-carotene.

It was observed that overexpression of *DcBCH1* had a greater effect on the accumulation of carotenoids in carrot taproot than overexpression of *DcBCH2*. We further obtained *DcBCH1* knockout mutant carrot, and found that the α-carotene content in the *DcBCH1*-knockout carrot taproot was slight higher, while the β-carotene content was significantly lower in comparison with the control carrot. In potato (*Solanum tuberosum*) tuber with both *CHY1* and *CHY2* silenced, the contents of β-carotene and total carotenoids increased, zeaxanthin content decreased; the expression of *Lut5* that encodes a β-ring hydroxylase was suppressed [45]. The contents of β-carotene-drevied xanthophylls, β-cryptoxanthin and zeaxanthin, were increased in *IbCHY-β* silenced sweet potato calli, storage roots, and leaves, possibly due to the up-regulation of the *IbP450* expression level, which is also known to have β-carotene hydroxylase activity, compensated for low level of *IbCHY-β* [46, 47]. In *A. thaliana*, the *b1* (*β-OHase 1*) mutant had a greater effect on carotenoid composition than *b2* (*β-OHase 2*), compared with the wild type plants; b2 could compensate for the loss of b1 enzymatic activity in *b1* mutant to a significant extent, and could partially compensate for the loss of b1 enzymatic activity in *lut1b1* double mutant [9]. The *DcBCH2* expression level in *DcBCH1*-knockout carrot was increased to varying degrees. CYP97A3, a hydroxylase belonged to cytochrome P450 class, hydroxylated both α-carotene and β-carotene, and had the main activity to α-carotene β-ring in *A. thaliana* [9]. A previous study found that the accumulation of α-carotene in orange carrot was caused by the loss of normal activity of CYP97A3 due to a frame shift insertion of *CYP97A3* [48]*.* In *DcBCH1* overexpression and knockout carrots, the expression level of *DcCYP97A3* was significantly decreased compared with control carrots; whereas the *DcCYP97A3* in *DcBCH2*-overexpressing carrot was increased. Zeaxanthin epoxidase (ZEP) catalyzes zeaxanthin to antherxanthin, which in turn generates violaxanthin [49]. Carotenoid cleavage dioxygenases (CCDs), including CCD and NCED, are the main enzymes in the enzymatic degradation of carotenoids, and carotenoid-derived cleavage products play important roles in plant developmental processes and response to abiotic stress [3, 50]. Compared with control carrots, *DcZEP* expression levels were significantly increased in both *DcBCH1* overexpression and knockout carrots, while the *DcNCED3* expression level was lower in *DcBCH1* overexpression carrot, higher in *DcBCH1* knockout carrot. Based on these results, it can be speculated that in *DcBCH1* knockout carrot taproot, DcBCH2 (increased expression level of *DcBCH2*) partially supplements the loss of the enzymatic function of DcBCH1, so that β-carotene was processed by hydroxylation and epoxidation reactions produce β-carotene-derived xanthophylls, which was cleaved under the action of CCDs, leading to the β-carotene content decreasing. DcCCD1 (DcCCD1a in this study) has been shown to cleave δ-carotene and β-carotene *in vitro* [51]. *DcCCD1a* transcript level was higher in the taproot of the *DcBCH1* knockout carrot than in the control carrot. The cleavage of β-carotene by DcCCD1a may also contribute to the lower β-carotene content in the *DcBCH1* knockout carrot taproot.

The activity of enzymes related to carotenoid metabolism is one of the important factors affecting carotenoid accumulation in plants. Expression of endogenous carotenogenic genes is often altered by changing the levels of biosynthetic intermediates in this pathway [6, 41]. In *CHY1* and *CHY2* simultaneously silenced potato tuber that contained increased β-carotene and total carotenoids contents, and decreased zeaxanthin content, the expression levels of carotenoid metabolism-related genes including *PSY1*, *ZDS*, *CRTISO*, *LCY-e*, *LUT1*, *LCY-b*, and *ZEP* were up-regulated [45]. In *lbCHY-β-*silenced sweet potato storage root, total carotenoids and β-carotene contents were increased by 2-fold and 16-fold, respectively; the expression levels of *PSY*, *PDS*, *ZDS*, *ZEP*, and *NCED* were lower than those in non-transgenic plant [46]. In transgenic tomato fruits hosting kiwifruit *AcBCH*, the β-carotene and lycopene contents were reduced, lutein content was increased; most genes of carotenoid metabolic pathway (*PSY*, *PDS*, *ZDS*, *CRTISO*, *LCYb*, *ECH*, *BCH*, *ZEP*, *CCD*, and *NCED*) were expressed at lower levels than control plants [41]. Compared with the respective control carrot, the expression levels of carotenoid metabolic pathway earlier enzymes encoding genes (*DcGGPS1*, *DcPSY1*, *DcPDS*, *DcZDS1*, and *DcCRTISO*) were slightly increased or not significantly changed in the taproot of *DcBCH1*-overexpressing carrot, while the increase was greater in *DcBCH2*-overexpressing and *DcBCH1*-knockout carrots. The increase in one carotenoid came at the expense of others [19, 52]. PSY is considered to be the key rate-limiting enzyme in the carotenoid metabolic pathway [53]. Studies have confirmed that induced changes in the abundance of carotenoid metabolites or their downstream products negatively feed back and regulate the transcriptional or protein levels of PSY [48, 54]. Based on these results, it can be speculated that overexpression of *DcBCH1* may tend to change the proportion of different types of carotenoids in carrot taproot, so it has little effect on the transcription levels of early enzyme-encoding genes in the carotenoid metabolic pathway; in *DcBCH1* knockout carrot taproot, the downstream metabolites of β-carotene were degraded by CCDs, thereby negatively regulating the rate-limiting step of the carotenoid metabolic pathway. When the upstream biosynthesis rate increases, it triggers the increase of expression levels of genes encoding other related enzymes in the metabolic pathway. Alteration of carotenoid accumulation by genetic engineering is also influenced by the level of carotenoid accumulation in the species itself [52, 55]. This may be one of the reasons why the changes of carotenogenic genes in different types of transgenic carrot taproots obtained in this study were different from those in transgenic materials of other species. Even though zeaxanthin content was increased in transgenic tobacco hosting *A. thaliana chyB*, this increase was not unlimited [56]. Carotene ε-cyclase (ECH/CYP97C1) interacts with BCH on α-carotene and catalyzes the biosynthesis of lutein [16]. The expression level of *DcECH* in *DcBCH1*-overexpressing carrot taproot was not significantly changed, whereas in *DcBCH2*-overexpressing carrot taproot was significantly higher, compared with control carrots. In *DcBCH1*/*DcBCH2*-overexpressing carrots, although α-carotene was reduced, the direct hydroxylated product lutein was not greatly increased (*DcBCH1-*overexpressing) or did not change significantly (*DcBCH2-*overexpressing), accompanied by the expression levels of *CCDs* (*DcCCD4*, *DcCCD1a*, and or *DcNCED3*) being significantly elevated in these two types of transgenic carrot plants. These results implied that the increased expression levels of these genes may maintain the stability of carotenoid metabolism by enhancing the cleavage of carotenoids. Previous studies have suggested that DcCCD1 may compete with β-carotene hydroxylase for β-carotene substrate in *in vitro* experiment [51]. Our previous results showed that DcCCD4 could cleave α-carotene and β-carotene [57]. It suggested there may be partly a degree of substrate competition between DcBCH and DcCCD, which may also be one of the factors affecting the changes of *DcCCD* transcript levels in different types of transgenic carrot taproots. In *DcBCH2*-overexpressing carrot, the transcription levels of *DcCCD1a* and *DcCCD4* were significantly increased, especially *DcCCD4*, speculating that the deepening of the competition relationship may cause changes in the levels of intermediate metabolites and thus lead to changes in the transcription levels of other carotenogenic genes. Due to the complexity of interactions and regulation between enzymes in the carotenoid metabolic pathway, further studies are needed to elucidate the regulation of carotenoid metabolic pathway by intermediate metabolites.

In summary, our results indicated that both DcBCH1 and DcBCH2 have hydroxylation effects on β-ring-containing carotene (α-carotene and β-carotene). DcBCH1 and DcBCH2 have functional redundancy. The enzymatic activity of BCH in carrot taproot mainly depended on the function of enzyme encoded by *DcBCH1* rather than *DcBCH2*, and these two DcBCHs can partially compensate for each other. The mutual compensation degree between DcBCH1 and DcBCH2 needs further analysis. Our results will provide a certain reference for further analysis of the metabolic mechanism of carotenoids and generation of materials with different types of carotenoids through genetic engineering.

## Materials and methods

### Plant materials and growth conditions

The carrot ‘Baiyu’ (‘BY’, nearly white) and ‘Kurodagosun’ (‘KRD’, orange) with different taproot color were used as experimental materials. Seeds of carrot were placed on moist filter paper to germinate. Then, the germinated seeds were transferred to flower pots and grown in a glass intelligent greenhouse of the State Key Laboratory of Crop Genetics and Germplasm Enhancement of Nanjing Agricultural University. The growth conditions of the glass intelligent greenhouse were set as 12 h light/12 h dark at 28°C. Carrot taproot was sampled at 85 d after sowing, and the samples were frozen and stored at −80°C immediately.

### Total RNA, genomic DNA (gDNA) extraction and gene cloning

We extracted total RNA and synthesized cDNA according to the previously described method [58]. gDNA was extracted from the samples using a DNAsecure plant kit (Tiangen, Beijing, China). The annotated BCH encoding DNA sequences (*DcBCH1* DCAR_020269, *DcBCH2* DCAR_014519) were searched in the published carrot genome [28, 59] and specific primers were designed (Table S1, see online supplementary material) to specifically amplify *DcBCH1* and *DcBCH2* from the cDNA of ‘KRD’. The full-length cDNA of *DcBCH1* cloned form ‘KRD’ was described in our previous study [58]. The full-length cDNA of *DcBCH2* was amplified by RT-PCR from carrot ‘KRD’ with the specific primers (Table S1, see online supplementary material). RT-PCR program parameters were 98°C for 3 min, 34 cycles (98°C for 10 s, 55°C for 30 s, 72°C for 15 s) and 72°C for 10 min. The amplification product was analysed by gel agarose electrophoresis and subsequently sequenced in Genscript (Nanjing, China).

### Bioinformatic analysis

The conserved domains were analysed using the NCBI database (http://www.ncbi.nlm.nih.gov) and the amino acid sequences of BCHs from other species were also downloaded from the NCBI database. DNAMAN 6.0 software was used to perform multiple sequence alignment of the BCHs amino acid sequences. The phylogenetic tree was generated from the amino acid sequences of BCH proteins by MEGA 5.0 using the neighbor-joining method [60, 61]. The chromosomal loci and the locations of exons and introns were defined using MapChart software and Gene structure display server tool (GSDS, http://gsds.cbi.pku.edu.cn), respectively, based on the carrot genomic database [62, 63].

### Subcellular localization analysis

The ORF of *DcBCH1* and *DcBCH2* without stop codon was PCR-amplified using specific primers (Table S1, see online supplementary material) from ‘KRD’ and fused with the enhanced green fluorescent protein (EGFP) gene in pSPYE vector to construct plasmid *DcBCH1-EGFP* and *DcBCH2-EGFP*, respectively. The *DcBCH1-EGFP* and *DcBCH2-EGFP* construct plasmids were introduced into *Agrobacterium tumefaciens* (strain GV3101) by electrotransformation, and transformed into tobacco (*Nicotiana benthamiana*) leaf cells using *Agrobacterium* infection method described previously [64]. After 5 d of *Agrobacterium* infection, the fluorescence signal of EGFP fusion proteins and red chloroplast auto fluorescence were observed and imaged using a LSM780 confocal microscopy (Zeiss LSM 780, Oberkochen, Germany).

### Overexpression vector construction and plant transformation

For the generation of *DcBCH1* and *DcBCH2* overexpression carrots, the full length of the *DcBCH2* was PCR amplified from ‘KRD’ using a pair of specific primers (Table S1, see online supplementary material) and cloned into the pCAMBIA1301 vector to yield the recombinant plasmid *35S:DcBCH2*. The *35S:DcBCH1* recombinant plasmid used was the same as in our previously study [58]. After the recombinant plasmids were verified by sequencing, they were introduced into *A. tumefaciens* (strain GV3101) by electroporation. The *35S:DcBCH1* and *35S:DcBCH2* constructs were transformed into orange carrot (‘KRD’) to generate stable transgenic carrots using *Agrobacterium-*mediated method, respectively [65]. Transgenic carrot was detected by PCR amplification and RT-qPCR analysis.

### Generation of CRISPR/Cas9-mediated *DcBCH1* knockout mutant carrot

Based on the published carrot genome database, the gDNA sequence of *DcBCH1* was obtained. Four target sequences in the *DcBCH1* were selected using the online tool CRISPR-GE (http://skl.scau.edu.cn/targetdesign/) for designing the sgRNA sequences. The four sgRNA expression cassettes containing four target sites driven by AtU3b, AtU3d, AtU6–1, and AtU6–29 promoter, respectively, were assembled and inserted into the corresponding site of the pYLCRISPR/Cas9Pubi-H binary vector to obtain the expression vector *DcBCH1-4xsgRNA/Cas9* according to the previously described method [65, 66]. The vector (*DcBCH1-4xsgRNA/Cas9*) was transferred into *A. tumefaciens* (strain GV3101) by electroporation. *DcBCH1* knockout mutant carrot was generated by the *Agrobacterium-*mediated method as previously described [65]. The gDNA was exacted from obtained transgenic resistant carrot plants and amplified the target fragment containing the target site using specific primers (Table S1, see online supplementary material) by RT-PCR, and then sequenced in Genscript (Nanjing, China) to detect mutations. Mutations that produced superimposed sequencing chromatograms were decoded using the online tool DSDecode [67].

### Carotenoid contents measurements

The samples were grated with liquid nitrogen and lyophilized in a vacuum dryer (Christ, Osterode, Germany). Approximately 50 mg of freeze-dried samples were extracted with 2 mL acetone, repeating this step until the samples were colorless and combined the extracts. The carotenoids extracts were detected using a Thermo UltiMate UHPLC system as in the previously described method [58]. Carotenoid species (α-carotene, β-carotene, and lutein) were identified in the extracts by comparison of retention times with standard samples. All data are quantified according to their respective standard curves. The specific carotenoid (α-carotene, β-carotene, and lutein) content was expressed as μg/g of dry weight (μg/g DW).

### Reverse transcription quantitative real-time PCR (RT-qPCR) analysis

RT-qPCR analysis was performed using SYBR Premix *Ex Taq* kit (TaKaRa, Dalian, China) as described before [64]. The primers used for gene expression analysis were referenced from previous study or designed using the Primer Premier 6.0 software (Table S1, see online supplementary material) [26]. The data were normalized to the expression of the *DcActin* and calculated according to the 2^*−*∆∆CT^ method [68, 69].

### Statistical analysis

The data were analysed using SPSS 20.0 software. The significant differences in gene expression and physiological parameter data between control carrot and transgenic carrot were analysed using the one-way analysis of variance (ANOVA) with Student’s *t*-test at the significance levels of *P* < 0.05 (^*^), *P* < 0.01 (^**^), and *P* < 0.001 (^***^).The significant differences in *DcBCH1* and *DcBCH2* expression between two carrot cultivars were compared using Tukey’s multiple range test (*P* < 0.05).

## Accession numbers

Sequence data from this article can be found in the NCBI database (http://www.ncbi.nlm.nih.gov) under the following accession numbers: *Pistacia vera* PvBCH2 (XP_031249306.1), *Petunia hybrid* PhBCH1 (QBC36226.1), *Ricinus communis* RcBCH2 (XP_002513654.1), *Prunus dulcis* PdBCH1 (BBG98323.1), *Vigna angularis* VaBCH2 (XP_017413495.1), *Benincasa hispida* BhBCH1 (XP_038907152.1), *Carica papaya* CpBCH2 (XP_021892139.1), *Herrania umbratica* HuBCH1 (XP_021285832.1), *Apium graveolens* AgBCH1 (QDC33551.1), *Juglans regia* JrBCH2 (XP_018809669.1), *Populus euphratica* PeBCH2 (XP_011035341.1), *Gentiana lutea* GlBCH1 (BAE92729.1), *Adonis aestivalis* AaBCH1 (ABI93208.1), *Rosa chinensis* RcBCH2 (XP_024191328.1), *Zea mays* ZmBCH1 (NP_001105865.2), ZmBCH2 (NP_001105907.1), *Punica granatum* PgBCH2 (XP_031383909.1), *Arabidopsis thaliana* AtBCH1 (NP_001320065.1), AtBCH2 (NP_001119420.1), *Malus domestica* MdBCH2 (XP_008343769.2), *Nicotiana tabacum* NtBCH2 (XP_016467042.1), *Solanum lycopersicum* SlBCH1 (NP_001234348.1), SlBCH2 (NP_001265981.1), *Vitis riparia* VrBCH2 (XP_034710058.1).

## Acknowledgements

The research was supported by National Natural Science Foundation of China (31872098; 32072563), Priority Academic Program Development of Jiangsu Higher Education Institutions Project (PAPD).

## Author contributions

A-S.X. and T.L. initiated and designed the research. T.L., J-X.L., Y-J.D., A-Q.D., and H.L. performed the experiments. T.L., J-X.L., and F-Y.Z. analysed the data. A-S.X. contributed reagents/materials/analysis tools. T.L. wrote the paper. A-S.X. and T.L. revised the paper. All authors read and approved the final manuscript.

## Data availability

All data supporting the findings of this study are available within the paper and within its supplementary materials published online.

## Conflict of interest

The authors declare that there are no competing interests.

## Supplementary data

Supplementary data is available at *Horticulture Research * online.

## Supplementary Material

Web_Material_uhac193
