# Supplementary material for: Differential hydroxylation efficiency of the two non-heme carotene hydroxylases: DcBCH1, rather than DcBCH2, plays a major role in carrot taproot
Source: Hortic Res. 2022 Aug 30;9:uhac193. doi: 10.1093/hr/uhac193 (PMC9630967; doi:10.1093/hr/uhac193)
Supplement: Web_Material_uhac193 [file web_material_uhac193.docx]

**Differential hydroxylation efficiency of the two non-heme carotene hydroxylases: DcBCH1, rather than DcBCH2, plays a major role in carrot taproot**

Tong Li^1, #^, Jie-Xia Liu^1, #^, Yuan-Jie Deng^1^, Ao-Qi Duan^1^, Hui Liu^1^, Fei-Yun Zhuang^2^, Ai-Sheng Xiong^1^,*

*1 State Key Laboratory of Crop Genetics and Germplasm Enhancement, Ministry of Agriculture and Rural Affairs Key Laboratory of Biology and Germplasm Enhancement of Horticultural Crops in East China, College of Horticulture, Nanjing Agricultural University, 1 Weigang, Nanjing, 210095, China*

*2 Key Laboratory of Horticultural Crop Biology and Germplasm Innovation, Ministry of Agriculture; Institute of Vegetables and Flowers, Chinese Academy of Agricultural Science, Beijing 100081, China*

^#^Contributed equally to this work.

*Please address all correspondence to: A.S. Xiong (xiongaisheng@njau.edu.cn)

---------------

Dr. Ai-Sheng Xiong

Professor

State Key Laboratory of Crop Genetics and Germplasm Enhancement,

College of Horticulture,

Nanjing Agricultural University,

Nanjing, 210095, China

Fax: 86 25 84396790

Email: [xiongaisheng@njau.edu.cn](mailto:Xiongaisheng@njau.edu.cn)


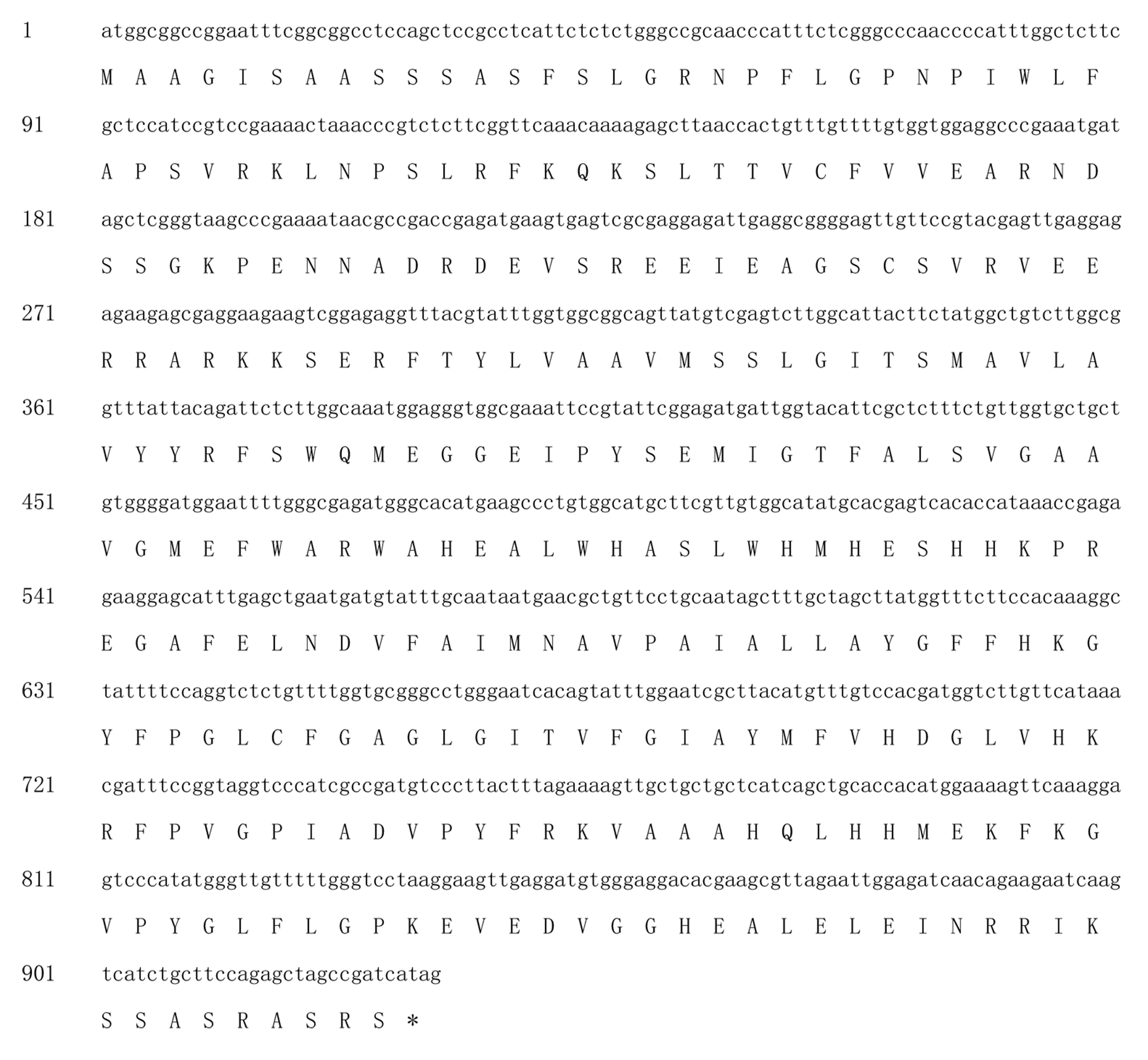


**Figure S1 Nucleotide acid and deduced amino acid sequence of *DcBCH1* gene from ‘KRD’**

* represents the stop codon. [58]


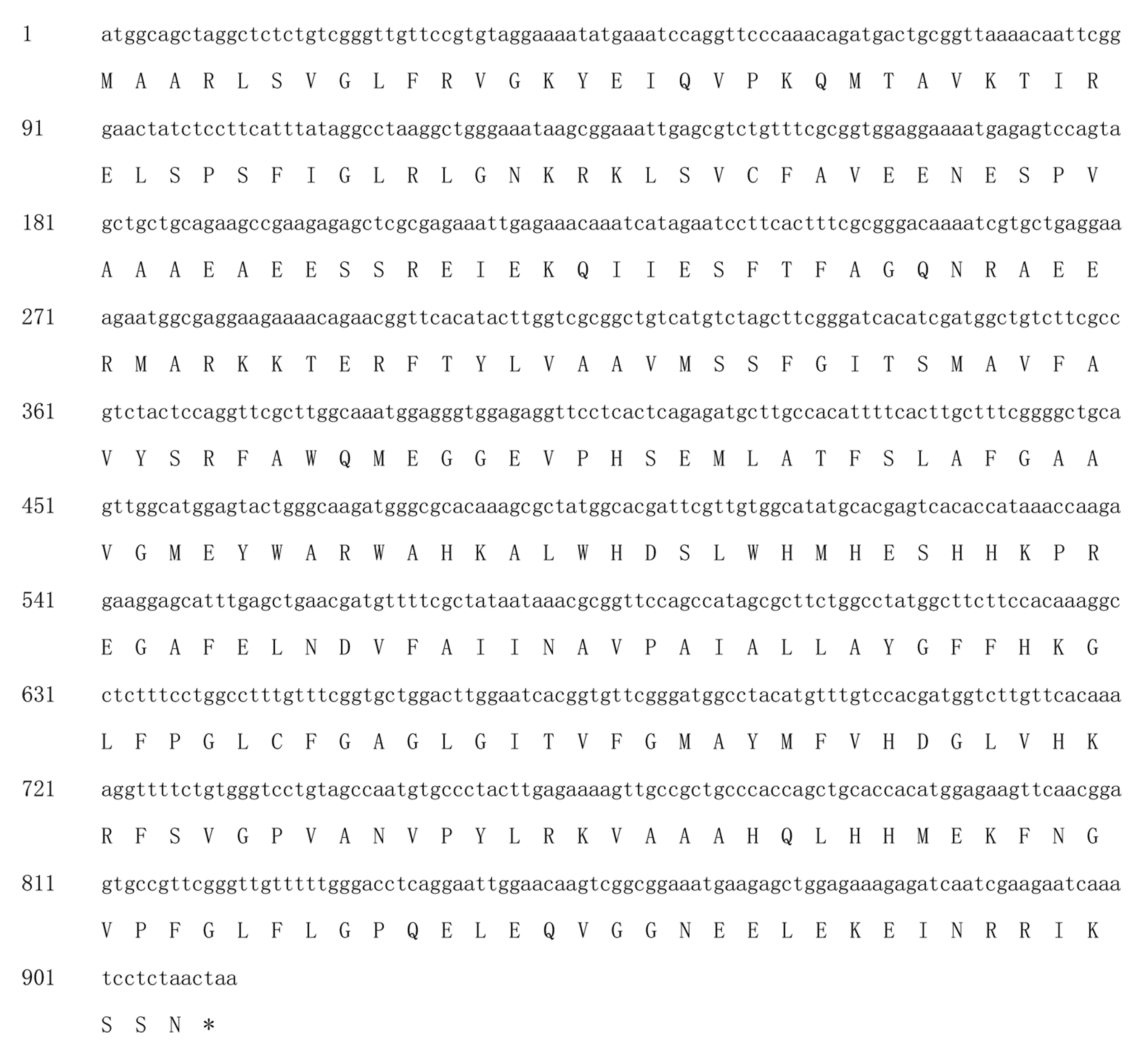


**Figure S2 Nucleotide acid and deduced amino acid sequence of *DcBCH2* gene from ‘KRD’**

* represents the stop codon.


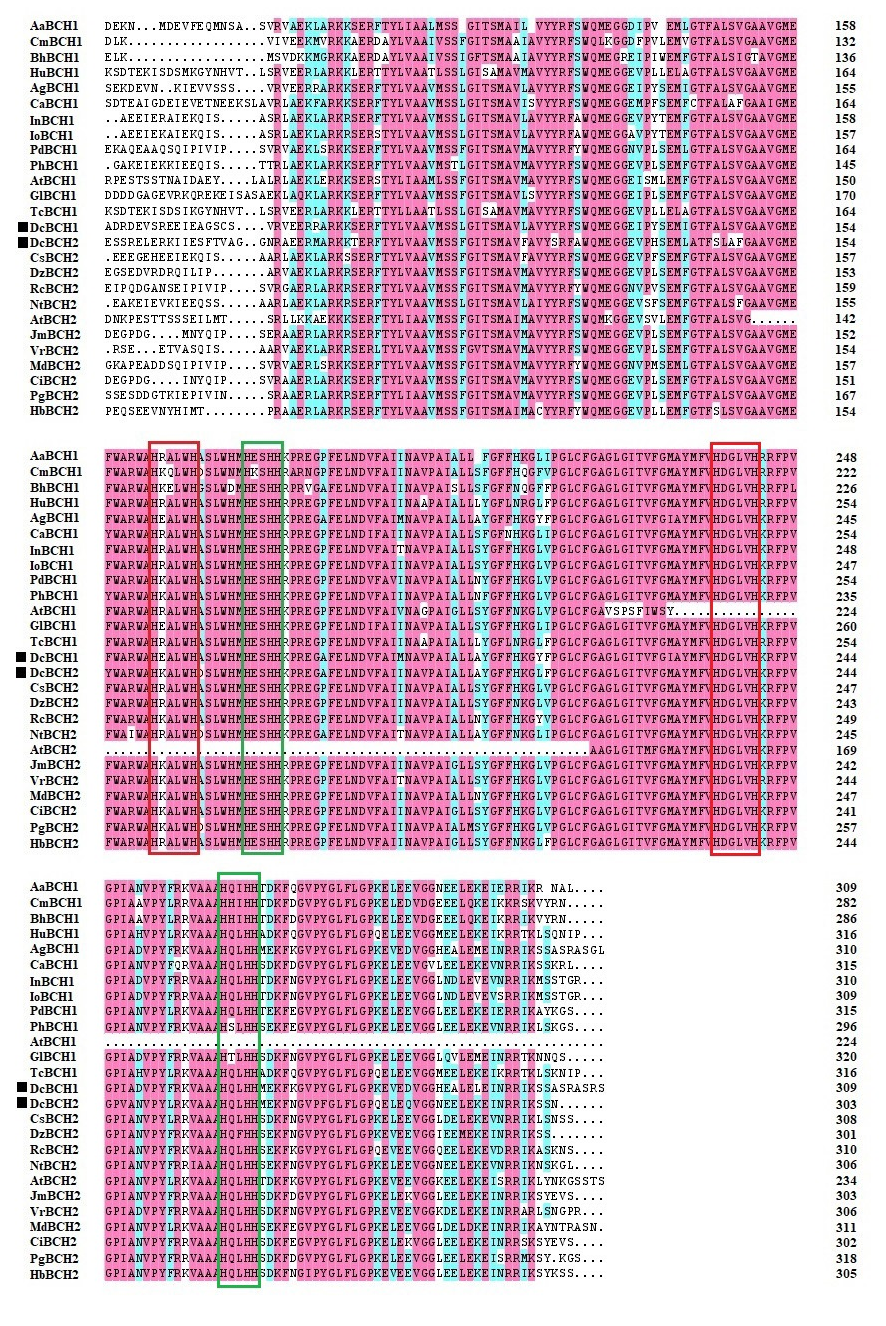


**Figure S3 Multiple alignments of DcBCH1 and DcBCH2 with BCHs from other species**

Note: Shown in the figure is the highly conserved region sequence. The red box indicates the “HX_4_H” domain; the blue box indicates the “HX_2_HH” domain.


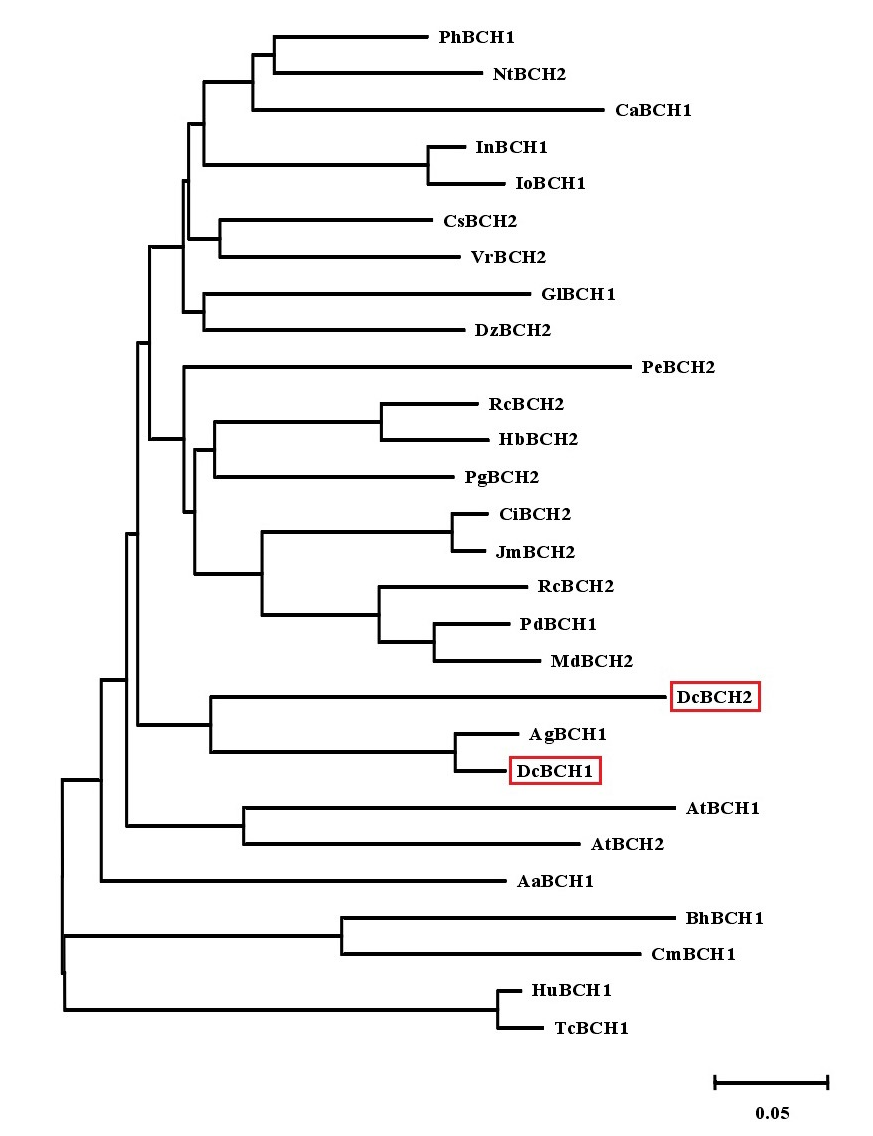


**Figure S4 Phylogenetic tree of DcBCH1 and DcBCH2 with BCHs from other species**

**Table S**1 Primer sequences used in this study

| Gene | Function | Forward primer (5'-3') | Reverse primer (5'-3') | Reference |
| --- | --- | --- | --- | --- |
| *DcBCH1* | Full lengths clone | ATGGCGGCCGGAATTTCGGCG | CTATGATCGGCTAGCTCTGGA | Li et al. 2021b |
| *DcBCH2* | Full lengths clone | ATGGCAGCTAGGCTCTCTGTC | TTAGTTAGAGGATTTGATTCT | - |
| *DcBCH1* | Overexpression vector construction | TTTACAATTACCATGGGATCCATGGCGGC  CGGAATTTCGGCG | ACCGATGATACGAACGAGCTCCTATGATCGGC  TAGCTCTGGA | Li et al. 2021b |
| *DcBCH2* | Overexpression vector construction | TTTACAATTACCATGGGATCCATGGCAGC  TAGGCTCTCTG | ACCGATGATACGAACGAGCTCTTAGTTAGAGG  ATTTGATTCT | - |
| *DcBCH1* | Subcellular localization | ACGGGGGACTCTAGAGGATCCATGGCGG  CCGGAATTTCGGCG | GCCCTTGCTCACCATGGATCCTGATCGGCTAGC  TCTGGA | - |
| *DcBCH2* | Subcellular localization | ACGGGGGACTCTAGAGGATCCATGGCAG  CTAGGCTCTCTG | GCCCTTGCTCACCATGGATCCGTTAGAGGATTT  GATTCT | - |
| *DcBCH1* | Mutation detection | ATGGCGGCCGGAATTTCGGCG | CATGCCACAGGGCTTCATGTG | - |
| *DcBCH1* | Mutation detection | TGGGGATGGAATTTTGGGCGAG | TGAACTTTTCCATGTGGTGC | - |
| *DcBCH1* | RT-qPCR | CTTGGCATTACTTCTATGGCTG | CGAATGTACCAATCATCTCCGAAT | Ma et al. 2017 |
| *DcBCH2* | RT-qPCR | CTCACTCAGAGATGCTTGCCACATT | TTCCGCCGACTTGTTCCAATTCC | - |
| *DcGGPS1* | RT-qPCR | GACGCTTCAGTCTCACTCCAAGA | GCAATACTAAGAAGAGGACGGACA | - |
| *DcPSY1* | RT-qPCR | ATACCAAACGGACAGGATCTAAG | CCTCATTCAACAAATTCCAGCT | Ma et al. 2017 |
| *DcPDS* | RT-qPCR | AAGTCAAGTTTGCGTTGGGTCTC | TCAGGTATGCCCTGCTTTCTCAT | Ma et al. 2017 |
| *DcZDS1* | RT-qPCR | CCCAGTTGGAGCACCATTACACGG | GGGCAAGGGCTACAGCATTTCTT | Ma et al. 2017 |
| *DcCRTISO* | RT-qPCR | GAAAAGCCTCAGACCCAGATGTC | TCTCCAACTCTTTATCAACACTC | Ma et al. 2017 |
| *DcLCYE* | RT-qPCR | GGATACTCAGTCTAAACTTGCCC | GCAGGACCACAACCAATAACCAC | Ma et al. 2017 |
| *DcLCYB* | RT-qPCR | GCAGGGTTAGCGGTAGCACAACA | TCCATAGCCTCAAACTCATCCAC | Ma et al. 2017 |
| *DcECH* | RT-qPCR | GCTTGCTGCTGGGCCGAGAGATTTT | GAGCCAAACAAGAACTCAGAAACCT | Ma et al. 2017 |
| *DcCYP97A3* | RT-qPCR | TGAGGAAGATGAGGAGGAGGAGAAG | GTGCTTGGCTACAGTAGGATCAGAA | - |
| *DcBCH1* | RT-qPCR | CTTGGCATTACTTCTATGGCTGTAT | CGAATGTACCAATCATCTCCGAAT | Ma et al. 2017 |
| *DcZEP* | RT-qPCR | CTTGATAAAGCATACAACCGAAGT | AAGTCCATGAATAACCGAGACAC | Ma et al. 2017 |
| *DcNCED3* | RT-qPCR | GCTTCGATGGCCTTAGATGCTGTC | GGTGGTGCTGGACTGGTTGTTC | - |
